# Emergency management: chemical burns

**Published:** 2018-11-09

**Authors:** Millicent Bore

**Affiliations:** 1 Cornea and Anterior Segment Specialist: Lecturer University of Nairobi, Kenyatta National Hospital, Nairobi, Kenya.

**Chemical injury causes severe corneal scarring, but this can be prevented by immediate irrigation of the eye.**

**Figure 1:**
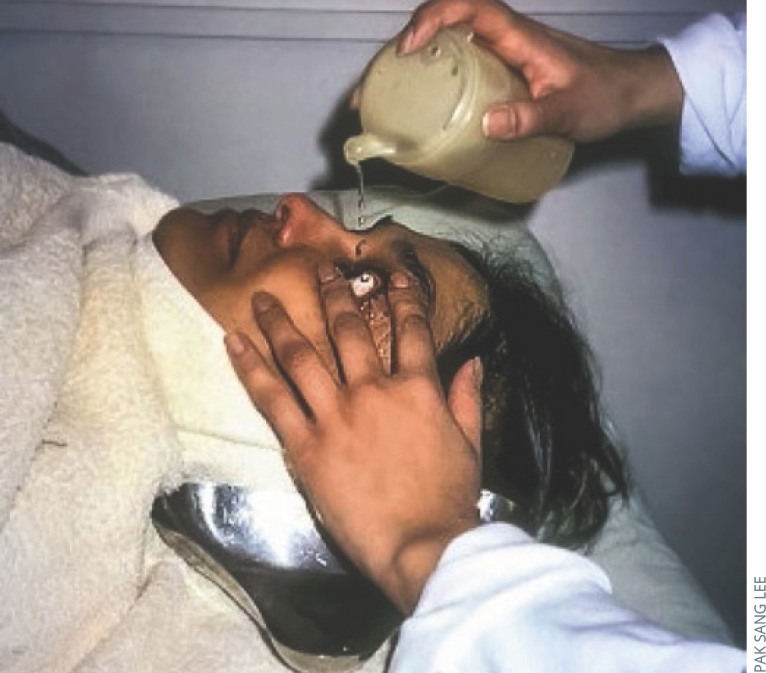
Irrigating the eye using saline solution and a feeding cup (if an IV set is not available). Irrigate for at least 30 minutes and take care not to contaminate the other eye.^2^,^3^

If a patient has suffered a chemical burn in the eye, immediate, high-volume irrigation is essential in order to wash out the chemicals and save her or his vision.^1^

1. Instil anaesthetic eyedrops and follow the protocol in the panel below to irrigate the eye for at least 30 minutes.^2^,^3^
2. After irrigation, give systemic analgesics and refer the patient to an eye specialist immediately.
3. If you are based in the community, refer patients to the nearest eye centre and tell them they must see an eye specialist very urgently.
4. Take a careful history and send your notes and the referral letter to the ophthalmologist, whether electronically or by hand. Make sure the patient knows where to go, and when. Call the ophthalmologist in advance so they know to expect the patient.

When taking the history, ask:

- When did the injury take place?
- Were the eyes rinsed out afterwards? For how long?
- What type of chemical splashed in the eye? Ask about the packaging
- Was she/he wearing eye protection?
- Accident or assault?

## Preparing for this emergency

Emergency management of patients with chemical burns requires preparation. It is important to have eye irrigation kits^2^,^3^ and protocols or standard operating procedures^2^,^3^ available that give clear guidance on what to do if a patient comes to a clinic or hospital with a chemical burn (see panels below). The kits should be easily accessible and everyone in the team must know where they are kept.

In a hospital, the personnel in the eye unit should practise and simulate the clinical scenario together with those from the emergency department because they are, in most cases, the patients' first contact when they arrive at the hospital. This preparation will save time, because everyone will know what is needed, where to find it and what role they will play. Time is of the essence, and a prompt response will limit damage to the eyes and save vision. This can only happen if personnel are well prepared.

In conclusion, immediate irrigation influences the final outcome favourably^4^ and improves prognosis. It is essential that all eye teams are trained and equipped to irrigate the eye.

**Eye irrigation kit**

- 2–3 litres of normal saline or Ringer's lactate (if not available, use clean water)
- Intravenous fluid (IV) giving set (or a large syringe or a small receptacle with a pouring spout), such as a feeding cup)
- Towel or gauze swabs
- A bowl or kidney dish
- Drip stand (if available)
- Local anaesthetic drops
- Eye lid speculum
- Retractors, if available
- Clean tissues
- Cycloplegic and antibiotic eyedrops
- An eye pad

**Protocol: irrigation following chemical burns**

1. Instil topical anaesthetic.
2. Insert lid speculum or use your fingers to gently hold the eyelids open.
3. Irrigate one eye at a time.
4. Tilt the patient's head towards the injured side to help avoid contaminating the other eye. Place a kidney dish ready to receive the fluid and use towels to enhance patient comfort. Ideally, use an IV giving set at full speed. However, in the absence of this, a clean container may be used.
5. Irrigate with normal saline or Ringer's lactate for at least 30 minute. Take care not to contaminate the other eye.
6. Evert the lid. Irrigate the front surface of the eye, including the fornices, slowly and steadily.
7. Ask the patient to move the eye in all directions while the irrigation is maintained.
8. Evert both the eyelids using retractors, then carefully assess for any particulate matter.
9. Remove particles from the ocular surface the corner of a folded tissue.
10. Instil cycloplegic drops (if available) and topical antibiotic eyedrops and pad the eye.
11. Give systemic analgesics.
12. Refer immediately.
